# The Treatment of Pulmonary Phthisis

**Published:** 1891-07

**Authors:** Thomas D. Coleman

**Affiliations:** Augusta, Ga.


### THE TREATMENT OF PULMONARY PHTHISIS—CONCLUDED.

BY THOMAS D. COLEMAN, A. B. M. D., AUGUSTA, GA.

It has been stated that the turpentine given off by the pine, converts O into ozone. Whether this be true or not, it is very certain that the air wafted from pine forests is extremely bracing. Added to this, the number of sunshiny days are so great that lawn tennis, riding, driving, and all out of door sports, can be enjoyed to the heart's content. Even after a heavy rain, it is necessary to remain in door scarcely more than an hour, for the drainage is so good and the soil of these hills so porous and sandy, that it soon absorbs even an unusual amount of rain.

One precaution which I would urge phthisical patients in all climates to regard, is the wearing of flannel from neck to toes Another is, that the exercise taken shall not be in excess of the patient's strength. Dr. Karl Von Ruck has presented an admirable paper on the dangers of overexertion. Patients are very apt to feel, when improvement begins, that they are capable of a greater amount of exertion than is actually possible with them. These, when they refuse to hearken to the advice of the physician, usually pay up for their heedlessness in relapses, and often with their lives.

### THE HYDROPATIC TREATMENT OF PHTHISIS.

This treatment is highly beneficial when carried out with diligence and discretion. The proportion of cases to which it is not applicable as a powerful adjuvant to other treatments of a hygienic and medicinal character, is very small. In itself it is inadequate, just as the administration of iron, or cod liver oil, or creosote alone is not all-sufficient for such a complex and obstinate malady. The hydropathic treatment simply fills one of the many requirements that are necessary for the most permanent and rapid repair that is possible. All life require a stimulus for healthy action. From the sensitive plant that simply requires the stimulus of the force and dimensions of a small insect in order to close in upon its prey—from the oyster that needs only the stimulation of a passing cloud over an otherwise cloudless sky, to make him rapidly shut himself within the thick walls of his shell-house, to man himself, there is always need of a stimulus in this world, where the struggle for existence is such a fierce one. It is not a single stimulus that is needed—the simple " make " or " break " connection of the galvanic current ; it is rather the interrupted though never-ceasing stimulation of the galvano-faradic battery. It is an undisputed fact that bodily activity is lowest in the very early morning. At the rising time, therefore, it is good for even the healthy to have the tonic benefits of a bath. How much more so is it with those debilitated by disease that they should have this stimulating effect—this general toning of all the vital organs. There are undoubtedly cases that cannot stand a cold bath at any time of day, but if these will begin at a temperature of lukewarm water and have a brisk rubbing down with a bath towel afterwards, it will be not only well tolerated, but will bring about a tingling and an amount of vitality as surprising as gratifying. It also induces a healthy action of skin that is much needed and so often lacking in phthisical patients. Where the lungs are involved with consolidations and cavities, and respiration is a difficult matter at best, it is imperative that the skin should be more than normally active, for greater work is thrown upon it. Winternitz, in his most admirable treatise entitled " Studien Fur Pathologie und Hydrotherapies der Lungen—Phthisies," says that " water or the temperature conveyed to it, is the best, simplest and most accessible means for strengthening and hardening a feeble body which is predisposed to catarrhs or colds. The simple rubbing of the entire surface immediately after rising from bed with a wet, cold cloth, accustoms the skin to sudden cooling, exercises the neuropruicular system of the peripheral arteries to prompt reaction, and acts as a thermic irritant centripetally on the central nervous system, and from thence acts eccentrically, stimulating the innervation and functions of respiration, circulation and digestion." It is well to begin with luke warm water and diminish the temperature 5 degrees to 1 degree every day.

MEDICINAL TREATMENT.—The medicinal treatment of phthisis has, up to this time, not been all that could be desired. Advance in this direction has been in the face of the greatest obstacles, and the disease has baffled the skill of one generation after another. Results have come slowly, and often been discouraging. But a brighter era is dawning and the treatment of this disease, while still very defective, is improving day by day, and the percentage of deaths constantly diminishing. But the extreme difficulty attending the treatment may be understood when we see the character of the organism against which we are battling. Filleau and Leon Petit affirm that: "The bacillus of tuberculosis, of all micro-organisms, is one of the most refractory to the action of the most destructive agencies. It maintains its virulence after lying for forty days in putrid sputum, and for one hundred and eighty six days away from the contact of air. It can live at temperatures between 86 and 104 degrees F. The most unfavorable conditions, though affecting its activity, do not compromise its existence, for it resumes its virulence whenever its surroundings become favorable." It is, no doubt, due to this fact that many of the cases of so-called cures finally die of pththisis.

COD LIVER OIL.—This oil, as is well known, is gotten from the livers of the cod—Gadus Morrhua—which lives at a great depth and in waters that are very cold, where the consumption of fat is a very slow process. This oil is fat that has undergone partial digestion. It is fat that has undergone digestion in the stomach and intestines and passed through the portal system to the liver, where it is stored up for the use of the animal. This fact makes it very much more valuable as a food than any other fat or oil with which we are acquainted. The emulsification of fats takes place in the small intestines when the alkaline juices of the liver and pancreas come in contact with the partly digested food (chyme) from the acid stomach. It causes neutral fats to take up a molecule of water, and having done this to split up into its corresponding fatty acid and glycerine. This is due to the presence of an easily decomposable fat splitting ferment, called steapsin by Claude Bernard. Almost all fats containfatty acids. When a fatty acid comes in contact with an alkali it forms a soap,which splits up the fat molecule and makes a permanent emulsion that can be absorbed by the blood. Now, cod liver oil is oil that has undergone this process in a large degree. It is more quickly dialyzable through animal membranes than any other fat or oil, and rises very much higher in capillary tubes that have been moistened with bile salts. These laboratory experiments, that can be repeated *ad libitum*, find confirmation in clinical experience, for there is no physician who has not observed a greater ease of assimilation than with any other oil. It is beyond all question the most concentrated form of food that can be obtained, and its chief good in this disease comes in the amount of nourishment that it affords the body. Besides its action in building up impoverished tissues, it has medicinal properties, i e., that of diminishing the cough and night sweats and increasing the appetite in a way not altogether understood. It contains a small trace of iodine, but iodine, combined with any other fat or oil, does not yield the same results. Its influence in aborting cases of incipient phthisis and inducing rapid improvement where the lesion is more marked cannot well be overestimated. Dr. Williams of England, one of the greatest authorities on phthisis that ever lived, gives a very instructive resume of his wide experience with and without the use of cod liver oil. He stated once "that whereas, forty years ago the average duration of life after the symptoms of the disease had been developed was only twenty-two months, it is now eight years." This statement rested on an experience extending over a period of forty years. His cases when they reached 12,000, were divided into periods of ten years each, and he gave the duration of life during the first, second, third and fourth decades. In the first decade, where no cod liver oil was used, the average duration of life was twenty-two months from the beginning of the disease to its termination. In the last decades where cod liver oil was employed, the period from the beginning to the end was eight and a half years. His testimony was that, unquestionably, the increase in time was due to the use of cod liver oil. Examples of this kind might be multipled indefinitely, but are unnecessary in a paper of this scope.

As to the preparations that are found to be most satisfactory, the pure Norwegian oil is considered the best, where stomachs will tolerate it; but, oftentimes, it happens that the pure oil cannot be retained, and then some of the various perfect emulsions are indicated. That made according to the American Pharmacopœia,—and also a recent preparation of maltine and oil, where you get, in addition to the palatability, a tonic effect from the maltine,—are readily tolerated, even by stomachs in a condition of unstable equilibrium. The difficulty with all emulsions is, that an inferior grade of oil or a diminished quantity, or both, may be used by uncrupulous compounders, without the knowledge of the physician or patient. For this reason, it is best, when possible, to prescribe the pure oil. Cod liver oil should always be given about half an hour after eating. When taken at this time it is more apt to be easily digested. Dose, from ʒ ii to ʒ vi. Cod liver oil should not be given when there is any great febrile action, for then all the normal secretions of the glands of the body are interfered with, and the assimilation of the oil made extremely difficult or impossible. Its use should also not be continued if there are constant eructations of it, hours after it has been taken, as this shows that it is not being properly assimilated. Attempts have been made to use other oils in place of cod liver oil, but, as a rule, no substitute has been found, and this, for reasons already given. Linseed oil has probably been more generally employed than any save cod liver oil; but only a few cases can take it and improve on it.

BEEF TEA AND BEEF EXTRACTS.

The reason for mentioning these is simply to show their usefulness. Liebig states that the greatest care is taken to exclude all fibrin gelaten, albumen and fat from his extract. He further adds that "its component parts do not give strength where there is none; that to extractives and salts is due all the value it possesses; that it is to be classed with tea and coffee; and that it neither economizes carbon for our temperature nor nitrogen for the sustenance of our tissues." Dr. Hassell has shown that 14 1-2 pounds of beef would be required to yield beef tea enough to supply the nitrogenous daily waste of one individaul, calculating that such waste amounts to 512 grains of urea and 21 grains of uric acid daily. Experiments made upon dogs have brought out the remarkable fact that they starve to death sooner when fed exclusively on Liebig's extract than when left unfed.

Milk is, of course, the one article of diet that should be forced as much as possible, since it is agreeagble to the palate of most persons and easy of assimilation. There is, in realty, scarcely an article of diet that can take its place. Taken in large quantity a certain amount of it must be stored up as fat, since it cannot easily be eliminated from the body. If the stomach will not tolerate the sweet milk, it may be sterilized, peptonized or pancreatized as the individual taste and digestion may demand. Buttermilk and koumyss are often well borne when other forms of milk are not. The process of "force feeding" is a rational and scientific plan. It has been prominently brought forward by Debove and Weir Mitchel, both of whom testify loudly in its favor. By this process food is taken at stated intervals, whether desired or not, then come periods of rest and exercise. When the anorexia is extreme the feeding is carried on through a stomach tube passed through the nose into the stomach.

ALCOHOL.—With phthisical as with all other patients, the indiscriminate use of alcohol is to be condemned. It should be prescribed only after a careful examination of the individual case, and then should be entered upon gradually and in moderation. When it is found that patients grow more bouyant under its use, the appetite fuller, and the night sweats diminish, then a little more latitude in its employment can be allowed. Beyond all question, alcohol is extremely beneficial in a vast majority of cases of pulmonary tuberculosis. It produces a warmth in the stomach that is grateful, and causes a desire for food.

The stimulation of the heart and increasing of the functional activity of the skin are also not to be disregarded. Its chief mode of action and its method of greatest good are to be found in the action on the central nervous system. This becomes stimulated after the introduction of alcohol into the system, and this in turn causes a general stimulation of all the organs of the body. Some authorities make it a routine practice in a majority of their cases to prescribe from a quart to a quart and a half of wine and one to two ounces of brandy daily. From this practice they claim to obtain most highly gratifying results. That it has any medicinal value beyond that already mentioned is not contended.

In cases with excitable heart action and tendency toward hæmoptysis, the stronger stimulants are, for most obvious reasons, contraindicated. If any should be allowed, it should be limited to beer or cider. It is better still in these cases, to take away all alcohol, and allow only a milk diet. Why is it that such relief is experienced when medicines that stimulate the heart, and those that cause dilatation of the peripheral vessels, are administered? It is because there is less work for the heart to accomplish, from the fact that the calibre of the smaller radicles is increased, thus affording the largest amount of blood to the greatest amount of aeration possible to the area of lung substance that functions. The law of the diffusion of gases is an important one, and bears materially on this point. It is the principle of dialysis applied to gases. If you take an animal membrane, and make with it a partition in a water-tight box, and put a strong salt solution in one side and pure water in the other, a diffusion will take place and in a few hours the water in the two sides will be equally salt.

Now, when you have the poorly oxidized blood in the capillaries of the lungs coming in contact with the freshly inspired atmospheric air there is an immediate surrounding of $CO_2$ and other deleterious products to the air and an oxygenation of the hæmoglobin of the blood, making oxyhæmoglobin, a very unstable compound which readily surrenders its O to the tissues in need of oxygen, and takes up $CO_2$ again, making $CO_2$ hæmoglobin and completing the cycle. Now, if you have a diminished capillary area in the lungs, due to consolidation and tubercular deposits, it is easily seen how a dilatation of the smaller arteries and a greater supply of blood to the capillaries will afford more extensive aeration. It is this which causes phthisical cases with diminished capillary areas to pant so for breath. It is the distress cry of the tissues for more oxygen. It oftentimes happens that a vaso-dilation is not in itself sufficient; there is a sluggishness of the blood currents due to diminution of the visatergo. The heart is weak and flabby and worn out; in this then is the important point in administering cardiac stimulants. Digitalis and strophanthus do stimulate the heart, but they also cause contraction of the peripheral vessels, so while you are giving the heart greater working capacity, you are increasing the work it has to do; but when you precede either by the use of nitroglycerine and drugs of this class, you have dilation of the peripheral vessels—a greatly strengthened heart and much less work to be accomplished, because peripheral resistance is diminished. Such drugs are always contraindicated where there is erosion of vessel walls and a tendency toward hæmoptysis. If the heart action absolutely demands their employment, they should be used only under the most careful scrutiny to see if unfavorable symptoms follow their administration.

Iron is indicated in these cases as in any other disease—where the anaemia is extreme, and not only the red blood corpuscles but the hæmoglobin is diminished—for iron is of itself a very important constituent of hæmoglobin. It is especially indicated after violent haemoptysis which has been followed by an attempt at strengthening the vessel walls by preceding its employment with doses of lacto-phosphate of lime.

QUININE.—Where there is marked hectic, quinine is given with decided benefit, in doses ranging from twenty to thirty grains per diem. It has a marked tonic effect and influences the night sweats, in some cases greatly diminishing them. The bisulphate and the hydrochlorate are by far the best preparations to employ, because of their much more rapid and greater solubility.

ANTIPYRETICS.—Those now employed are nearly all members of the coal tar series. Those most generally employed are antipyrine, acetanilid and phenacetine The drugs of this class are very efficacious in adding to the patient's comfort. They not only act as antipyretics and analgesics, but some observers claim an actual diminution in the night sweats and an improvement of the appetite.

In the night sweats that are very exhausting, atropia, in doses ranging from 1-240 grain to 1-160 grain, may be given. When this is not effective, it will be found that agaricin, in doses of 1-6 of a grain, will avail. Any of the anhidreotics may very well be supplemented by sponging with equal parts of alcohol and water.

PHOSPHOROUS.—In the form of hypophosphite of lime and soda, phosphorous was recommended nearly half a century ago, on the supposed ground that it diminished tissue waste and favored cell formation. It seems to act in some cases favorably, but in a majority of cases its efficacy is nil.

CREOSOTE.—This is an extremely complex substance, the chemists tell us, and it is as yet impossible to state whether the beneficial results in pulmonary phthisis are due to the complex whole or one element constituting it. The beech wood tar creosote is what is universally employed. The fact of its complex nature makes any one test for its presence out of the question. Creosote is the one remedy that has been tried by the greatest number of observers, and it has been found to be a very valuable remedy in phthisis. In looking over a large number of hospital reports one cannot but be struck by the universality of its employment and its equally good results. The use of creosote in phthisis is not entirely new. Dr. Thompson, in his clinical lectures on pulmonary consumption more than thirty years ago, gave a formula that was extremely good, and consisted of—

| | | |
|---|---|---|
| ℞. | Cod liver oil, - - - - | ℥ iss. |
| | Creosote, - - - - | gtt. iv. |
| | Tragacanth powder, - - | ʒ ii. |
| | Anise seed water, - - - | ℥ iss. |

Dose, ℥ i. t.c.d.

Guttmann has shown that tubercle baccilli will not grow in solutions of the strength of 1 to 2000 creosote, while cultures are but feeble in a concentration of 1 to 4000.

Dr. Robinson states that it is most efficacious in the first stage. He states that it "not only lessens or cures the cough, diminishes, favorably changes and occasionally stops the sputum, and relieves the dyspnœa in very many instances; but it often increases the appetite, promotes nutrition, and arrests night sweats." When given in doses too large, it upsets the stomach and bowels, and it is also claimed that it intensifies any renal complication that may exist. Summerbrodt is a believer in heroic doses, and takes the position that the blood may be so charged with it as to antagonize the development of tubercle bacilli. He has given as much as gr. xii daily. Burlureaux uses creosote in large doses, utilizing the pressure of the liquid to facilitate its absorption. A reservoir at a suitable height is used, communicating by means of a rubber tube with a perforated needle. The fluid comes down slowly and gradually for several hours, and penetrates into the cellular tissues. The solution employed is the following:

| | | |
|---|---|---|
| ℞. | Pure creosote, - - - | 10 gms. |
| | Pure olive oil sterilized, - | 150 gms. |

The place of injection was at the posterior part of the thorax, near the spine of the scapula. The injections do not cause local mischief when the oil is properly sterilized by boiling. In a space of time, varying from five to ten minutes, the characteristic odor of creosote is on the patient's breath, and this lasts for twelve hours. The injections are made every second day. Very gratifying results are claimed from this treatment.

Dr. W. H. Flint, a most acute observer, has made a valuable report before the New York Clinical Society. This consisted of a list of 73 cases, divided into three classes:

1. Those in which creosote inhalations alone were employed.

2. Those in which the drug was administered both by inhalation and by the stomach or the rectum.

3. Those in which the drug was given only by the stomach or rectum. None of these methods furnished invariably the best results. The inhalation method was the most successful for patients whose gastro-intestinal tracts were diseased, while the other methods were more satisfactory in cases whose digestive organs were in fairly healthy condition. A mixture of equal parts of alcohol, creosote and chloroform is used in inhalers. Time of inhalation varied from q. h. to q. 5 h.

For the administration by the mouth or rectum, an emulsion was used, consisting of cod liver oil 40 parts and mucilage of acacia 60, each drachm containing 2 minims of creosote. In suitable cases the emulsion was given q. 2 h. and the dose increased to the point of toleration, which was usually 10 to 12 minims per diem. When the creosote emulsion was shaken up with milk it was least irritating to the stomach.

Sommerbrodt, in 5,000 cases, gave it, and is convinced that it does a great deal more than improve the digestion.

℞. Creosote bitumini fage, - - grs. lxxvi.
Balsomi, - - - - - fl. ℥ viss.
Excipientes amari qs. ft. in pil ad c.

Sig: Dose, one pill on 1st day, 2 on the next, 3 daily for one week, and 4 daily for next week, etc., until 9 pills of creosote a day are reached.

Forms of inhalers are very varied, and need not be described. In using the one which consists of a mask with sponge in it to go over the nose, care is necessary in order to not blister that organ, a result which I have seen occur many times.

Guiacol is of very recent use, but is affording fair results. It seems to be far less irritating to the stomach than creosote. Its action is apparently anti-catarrhal. The claim is made that its efficiency is as great as that of creosote, but it will take time to verify such a claim. Eucalyptol, thymol and menthol all have their advocates; but their application is limited, to say nothing more.

Gases under pressure were at one time greatly in vogue, and pneumatic cabinets of every conceivable design and degree of complexity were constructed. Gases, under varying conditions of pressure, were employed. The employment of these has now been abandoned, in the face of more enlightened treatment. The men who contend for the virtues of such treatment are few, indeed. The large pneumatic cabinet which was put into the Presbyterian Hospital, in New York City, at such great cost, is now used, or was, at least, as a dark room for developing photographs of such specimens as may be desired by the house staff and attending physicians. The inhalation of oxygen in some cases, especially where much of the normal pulmonary area has been destroyed, is unquestionably good, but is not commensurate with the amount of trouble entailed, and in many cases is not obtainable. This whole method of treatment has been unsatisfactory, and is in nowise comparable to the pure air of a bracing mountain climate.

The inhalation of sulphurous acid is another method that has been employed in the treatment of phthisis, but, according to the investigations of Darieux, it rarely causes the amelioration of symptoms, and never the disappearance of bacilli from the sputum. Hydrofluoric acid inhalations have likewise been proven useless, and have been abandoned. Carbonic acid gas has been used, and one mode of its employment is curious, to say the least. Hugo Weber describes it as consisting of administering to a patient a teaspoonful of bicarb of soda, before meals, and following it with a wine-glass of water, containing twelve drops of hydrochloric acid. This generates about two hundred and seventy CC of $CO_2$, which is gradually absorbed and exhaled by the lungs.

Gaseous enemata in the treatment of phthisis were originated by Dr. L. Bergeon, formerly professor in the medical school at Lyons, France. It consists of introducing into the rectum $CO_2$ and $H_2S$, under pressure, and has been well described by Cohen, of Philadelphia.

The use of therapeutic agents for the cure of phthisis per rectum, is to-day entirely abandoned, according to such high authority as Dujardin Beaumetz.

HOT AIR INHALATIONS.—Louis Weigert, of Berlin, gets the credit of having introduced this method for the cure of phthisis. It is unnecessary to reproduce, in detail, all that he claimed for the treatment. Suffice it to say, that he claimed to obtain the most marvelous results. Reasoning on an erroneous theory for a major premise, a great many substantial men, observers, were led to accept the treatment as rational. Soon it reached the dimensions of a fad, and the journals were full of the Weigert cure of phthisis by inhalations of hot air. History repeats itself in the medical world, as elsewhere; and so it was with this as with the riddle that King Charles of England propounded to his scientists. He asked them why it was that when a live fish was placed in a tub of water it did not weigh any more than before the fish was put in the whole tub of water. They puzzled their brains for weeks to explain why it was that a live fish did not weigh anything in a tub of water. Suddenly it occurred to one of them that it would be a good thing to try it, and he was rewarded by finding that the tub of water with the fish weighed just as much more as the weight of the fish. Now, this is the history of the hot air treatment. Physicians accepted it without questioning, and patients were discomforted in undergoing the treatment, until it occurred to some men to really see how much of the hot air reached the lungs. During a service as assistant in physiology in the medical department of the University of the City of New York, I had the pleasure of assisting Dr. W. Gilman Thompson in a series of experiments on dogs and cats, to see just what the temperature was when it reached the lungs, and if it were sufficient to affect the life processes of the bacilli. The Weigert apparatus employed did not afford heat enough, so a coil of gas piping was used, through which air was forced by a bellows. The coil was heated to a white heat, and through this the air that the animal was to breathe was forced. A thermometer was placed in the muzzle at the nose, and one in the trachea, and in several instances, one in the lung substance.

The results gotten did not at all bear out the claims of the advocates of this treatment. It is surely difficult to understand how any one could believe that air hot enough to destroy bacilli, and not induce serious complications, could reach the lung substance. Dr. Thompson's interesting conclusions are as follows:

1. Continuous inhalations of air, heated and at a temperature of from 200 degrees to 300 degrees at the nose, do not raise the temperature of the lung at all in some cases, even when maintained for one or two hours.

2. In other cases if continued for an hour or more there may be a slight rise in temperature of from 2 degrees to 3 degrees due to various causes other than entrance of hot air.

Mosso and Rodelli have also made experiments on dogs. They inserted a thermometer into the trachea of a dog and forced it to breathe heated air. They found that directly under the larynx the temperature had already fallen from 160 degrees C to 38 degrees C. They used Weigert's apparatus. They showed also that the exhaled air, being saturated with moisture, is of the same temperature, whether inspired at 130 degrees C or 18 degrees C.

The testimony from clinical experience is equally as decided.

Troudeau, at the Association of American Physicians, gave his conclusions as follows:

"1. The therapeutic value of hot air inhalations in phthisis is doubtful.

"2. The evidence obtained by bacteriological study of the cases recorded does not confirm the assumption that inhalation of heated air can prevent the growth of the tubercle bacillus in the lungs of living individuals or diminish their virulence."

With this, I think, we can close up the volume of hot air treatment in phthisis.

In the article of Dr. W. G. Thompson, referred to above, will also be seen the effects obtained on animals breathing cold air. The absurdity of its employment with any hopes of checking hæmoptysis ought to be patent. It must of necessity be absolutely useless, and in my opinion harmful. There is nothing at this time that can take the place of rest in a recumbent position, hypodermics of morphia and ergot and the use of the ice pack, in the treatment of hæmoptysis.

THE KOCH TREATMENT.

The greatest interest in a paper on this subject and at this time will unquestionably centre in the attitude taken on the Koch treatment. To give a concise summary of the attitude of the highest medical authorities on this subject is an extremely difficult thing to do in the time allowed for this paper. Koch's "Tuberculin," as his lymph has been styled, consists of a glycerine extract derived from a pure culture of tubercle bacilli. "This extract," according to Koch, "contains not only the effective substance derived from the bacilli, but such other substances, consisting of salts, coloring material and extractive matter, as would be soluble in a 50 per cent. sol. of glycerine." "The effective matter is precipitated by alcohol, in which it is soluble, and can be isolated from other substances in a comparatively pure and concentrated form, and with increased potency." "It may be," he adds, "impossible to explain the manner in which this remedy exerts its specific influence upon tuberculous tissue." It is doubtless unfair to pass judgment upon Dr. Koch's life labor on such short trial; but to the candid observer, after reviewing the history of this treatment and all the reports on the same, it cannot but seem that Koch was forced to give out his discovery to the world prematurely. That some such material, with the qualities claimed by Dr. Koch for his "tuberculin," will ultimately be found, I have no doubt, but up to this time the results have been unsatisfactory and disappointing. It is to be hoped, however, that so astute a scientist as Dr. Koch will not rest satisfied until he has found this magic boon to mankind, and that generations may yet "rise up and call him blessed."

The effect of "tuberculin" on lupus, a tuberculous skin manifestation, seems to be all that has been claimed for it. This result is fully attested to by physicians in this country as well as abroad. The injections of the lymph are made with a Pravaz syringe. A 10 per cent. watery solution of the "tuberculin" is used, and the dose of this is 0.1 to 0.01 cc. The largest dose recorded up to this time was given by Dr. Levy at his sanitarium, and was 0.7 cc, after which the patient was comatose for 48 hours.

I will close this brief account of Koch's "tuberculin" by the following piece of wholesome advice, given by an intelligent observer, who says: "That it is dangerous to inject just any case with incipient phthisis. Not only the lungs, but the larynx of every case should be very carefully examined, for if there should be any tuberculous disease the lymph will find it, and may cause such an infiltration and œdema of the soft tissues as to necessitate a tracheotomy."

Grancher and Martin claim to have discovered a specific for pulmonary phthisis, and that their method, whatever it may consist of, was deposited, sealed, in the Paris Academy of Medicine, as early as November 19th, 1889.

Leibrich's remedy is still on trial, and has the endorsement of some of the best men in France and Germany. His remedy consists of the following: 0.2 grains of pure cantharadin and

0.4 grain of potassic hydrate in 20 cc. of water. He uses the remedy by subcutaneous injection, beginning with not more than one-fiftieth part of a decigram of the solution. The dose is then slowly increased until one or two decimilligrams have been attained. The drugs appear to affect diseased tissues only.

As to injections of antiseptic fluids into the lung substance, they have not been attended with results that could in any sense be called satisfactory.

And so the question still remains in statu quo, but let us trust that the goal may yet be reached, and humanity freed from the fearful ravages of this dread disease.

